# Nutrition Risk is Associated with Leukocyte Telomere Length in Middle-Aged Men and Women with at Least One Risk Factor for Cardiovascular Disease

**DOI:** 10.3390/nu11030508

**Published:** 2019-02-27

**Authors:** Melissa Ventura Marra, Margaret A. Drazba, Ida Holásková, William J. Belden

**Affiliations:** 1 Division of Animal and Nutritional Sciences, West Virginia University, Morgantown, WV 26506, USA; madrazba@mix.wvu.edu; 2 Office of Statistics, West Virginia University, Davis College of Agriculture, Natural Resources and Design, West Virginia Agriculture and Forestry Experiment Station, Morgantown, WV 26506-6108, USA; iholaskova@mail.wvu.edu; 3 Department of Animal Sciences, Rutgers, The State University of New Jersey, New Brunswick, NJ 08901-8554, USA; beldenwj@sebs.rutgers.edu

**Keywords:** telomere length, diet quality, dietary screening tool, Mediterranean diet

## Abstract

Poor diet quality has been associated with several age-related chronic conditions, but its relationship to telomere length, a biological marker of cellular aging, is unclear. The purpose of this cross-sectional study was to determine whether overall diet quality was associated with relative leukocyte telomere length (rLTL) in a sample (*n* = 96) of nonsmoking middle-aged adults in Appalachia with at least one risk factor for cardiovascular disease. Diet quality was assessed using the Healthy Eating Index (HEI-2015), the alternate Mediterranean diet score (aMed), and the Dietary Screening Tool (DST). Peripheral rLTL was measured by quantitative real-time polymerase chain reaction. The associations between potentially confounding sociodemographic, lifestyle and health-related factors and the first and fourth rLTL quartile groups were examined using Chi-square or Fisher’s Exact tests or logistic regression. The relationships between diet quality index scores and rLTL as a continuous variable were analyzed using simple linear regression and multivariate linear models, analogous to linear covariance analyses. The rLTL ranged from 0.46 to 1.49 (mean ± SEM was 1.02 ± 0.18). Smoking history, income level, and cardiovascular health (Life’s Simple 7) were associated with the lowest and highest quartiles of rLTL and were used as covariates. In adjusted and unadjusted models, participants considered “at nutrition risk” by the DST were more likely to have shorter rLTL than those “not at risk or at potential risk” (*p* = 0.004). However, there was no evidence that adherence to the 2015–2020 Dietary Guidelines for Americans or to a Mediterranean diet was associated with rLTL in this sample. Intervention studies are needed to determine if improving the diet quality of those at nutrition risk results in reduced telomere attrition over time.

## 1. Introduction

Telomeres are DNA-protein caps at the end of chromosomes that maintain genome integrity; the length of the telomere is considered a marker of cellular aging [1]. In somatic cells, telomeres shorten with each cell division [2,3] until they reach a critical length at which point cells stop dividing and become senescent [4]. Cellular senescence reduces tissue repair capacity and induces a proinflammatory state contributing to age-related disease [5]. Shorter telomeres have been reported in individuals with a range of age-related conditions including cardiovascular disease (CVD) [6,7], obesity [8], type 2 diabetes [9,10], and hypertension [2], often independent of chronological age. Although it is not clear if telomere shortening is a cause or a result of chronic disease states, it has been postulated that reducing the rate of telomere shortening could prevent age-associated diseases [11], thereby improving health span.

While telomere length (TL) at birth is genetically determined [12], the rate of telomere shortening over the life span is thought to be influenced, in part, by lifestyle factors including diet [13]. Single dietary components and food groups characteristic of healthy eating patterns such as a Mediterranean style diet and the Dietary Guidelines for Americans (DGAs) have been associated with longer TL in some cross-sectional studies. For example, individuals with higher intakes of dietary fiber [14], nuts and seeds [15], and fruits and vegetables [16] and lower intakes of saturated fat [17], sugar sweetened beverages [18], and processed meats [19] were reported to have longer TL than those with poorer intakes. A systematic review, however, concluded that while intakes of fruit and vegetables appeared to be supportive of longer TL, studies on other individual foods and nutrients were inconsistent [16]. Studies that focus on single nutrients and foods may yield inconsistent results and flawed conclusions as they do not account for the cumulative, synergistic, and antagonistic effects of overall diet. Therefore, studies that compare overall diet quality may be more informative to TL than single food groups or components.

Overall diet quality has been compared to TL in a two large cohort studies in the United States (U.S.). One study analyzed data from the Nurses’ Health Study and found that healthy middle-aged and older women with a greater adherence to a Mediterranean diet measured by the Alternate Mediterranean Diet (aMed) metric had longer telomeres than those with poorer adherence [13], but TL was not associated with individual components of the diet. A second study, a large U.S. population-based study, used data from the National Health and Nutrition Examination Survey (NHANES) to compare the adherence to multiple evidence-based dietary patterns to TL [20]. Results showed that those with better adherence to the DGAs, the Mediterranean diet, and the Dietary Approaches to Stop Hypertension (DASH) eating plan, measured by the Healthy Eating Index (HEI)-2010, aMed and the DASH diet scores respectively, were all associated with longer TL in healthy women, but not men. Adherence to these evidence-based dietary patterns have also been associated with lower rates of CVD risk and mortality among older adults [21], but their associations with TL in subpopulations at CVD risk is largely unknown.

Appalachia represents a health disparate region in the U.S. West Virginia (WV), the only state that lies 100% within the Appalachia region; that has the highest rates of CVD-related conditions such as obesity, type 2 diabetes, and hypertension [22]; and the lowest rates of adequate fruit and vegetable intakes in the nation [23]. The purpose of this study was to determine whether overall diet quality including adherence to the DGAs and a Mediterranean diet was associated with TL in a middle-aged Appalachian population after adjusting for confounding sociodemographic, lifestyle, and health-related variables. We hypothesized that diet quality scores would be independently and positively associated with rLTL.

## 2. Materials and Methods

### 2.1. Study Design and Participants

This cross-sectional study evaluated the relationship between diet quality measured using three metrics (HEI-2015, aMed and the Dietary Screening Tool (DST)) and relative leukocyte telomere length (rLTL) in a non-smoking middle-aged population in West Virginia. Participants were 45- to 64-year-old adults recruited from two counties by word-of-mouth and community advertising. Exclusion criteria included current smokers; diagnosis of cancer, kidney, heart or liver disease; surgery 6 months prior; anti-inflammatory or anti-coagulant medications; major diet/appetite change 3 months prior or abuse of alcohol or other substances. The study protocol (#1507753607) was approved by West Virginia University’s Institutional Review Board, and all participants provided informed consent. Participants were given a $100 gift card for participation.

### 2.2. Diet Quality and Nutrition Risk Assessment

Dietary intake was assessed using three 24-h recalls collected and analyzed using Nutrition Data Systems for Research (NDSR) software version 15 (2015) developed by the Nutrition Coordinating Center (NCC), University of Minnesota, Minneapolis, MN, USA. Interviews were conducted over the telephone by NCC- trained research personnel using the software’s integrated multiple-pass method on two weekdays and one weekend day. Diet quality was assessed using two diet quality index scores, the HEI-2015 and the aMed index score, in addition to a nutrition risk screener, the DST.

To obtain HEI-2015 index scores, NDSR food and nutrient output files were first transformed into HEI-2015 component variables using NDSR’s unpublished guide [24]. Then, the simple HEI scoring algorithm method for multiple days of intake data was applied [25]. A score was calculated for 13 diet variables: nine adequacy components (total fruit, whole fruit, total vegetables, greens and beans, whole grains, dairy, total protein foods, seafood and plant protein, and fatty acids) and four moderate components (refined grains, sodium, added sugars and saturated fats) using a density approach [26]. The 13 component scores were summed for a total HEI score ranging from 0–100, with higher scores indicating better adherence to the 2015 DGAs.

The aMed diet score, created based on intake patterns associated with the risk of chronic disease, was used to assess adherence to a Mediterranean style diet [27]. The NDSR output was used to determine intake values for each of the nine variables making up the index: eight desirable components (whole grains, vegetables, fruits, nuts, legumes, fish, the ratio of MUFAs to PUFAs, and alcohol), and one undesirable component (processed and red meat products). The component values were energy adjusted using a density approach and the intakes were compared to the sex-specific median from our sample population. Scores above the median for desirable components, and below the median for the undesirable component, were assigned 1 point; otherwise 0 points were assigned [28]. The nine component scores were summed for a total aMed score ranging from 0–9 with higher scores indicating better adherence to the Mediterranean diet [27,28].

Unlike the HEI-2015 and aMed index scores which are based on intakes from multiple 24-h recalls, the DST is a survey instrument made up of 24 questions about food intake and behaviors and one question about dietary supplement use [29]. The screening instrument was previously validated in this sample to assess overall diet quality and to detect nutritional risk particularly as it relates to fruit and vegetable intake [30]. For this study, the questions were asked on the online survey and the scoring algorithm was applied based on participant responses. The scores were summed for a total DST score ranging from 0–105 points with higher scores indicating better overall diet quality. Participant scores were categorized as being “at nutrition risk” (DST scores < 60) or “not at risk/at potential risk” (DST scores ≥ 60) [30].

### 2.3. Relative Leukocyte Telomere Length

A fasting venous blood draw was performed by a trained phlebotomist at the in-person session. Blood was collected in vacutainers containing ethylenediaminetetra acetic acid (EDTA). Whole blood was apportioned into 1 mL micro-centrifuge tubes and placed on dry ice for transport to West Virginia University where it was frozen at −80 °C until shipped for analysis. Genomic DNA from the whole blood was isolated from the de-identified and coded samples using a Zymo Research Quick-gDNA MiniPrep kit following the manufacture’s guidelines. rLTL was measured from the DNA by real-time quantitative polymerase chain reaction (qPCR) based on a published protocol with minor modifications [31]. Briefly, the rLTL was calculated as the ratio of the telomere repeat copy number to a single-copy control gene (T/S ratio). For each sample, the concentration of purified DNA was measured on a NanoDrop and diluted to 2 µg/mL in 96-well microtiter plate in 10 mM Tris-HCl, 0.1 mM EDTA, and pH 7.5. Then, 4.0 ng of DNA was used in a qPCR reaction with a SYBR Green Master Mix (Applied Biosystems). The concentration of the oligonucleotides used to amplify the telomere repeat was 270 nM for TelF (5′ GGT TTT TGA GGG TGA GGG TGA GGG TGA GGG TGA GGG T 3′) and 900 nM for TelR (5′ TCC CGA CTA TCC CTA TCC CTA TCC CTA TCC CTA TCC CTA 3′). The single copy gene, *Eif2a* was amplified using an oligonucleotide concentration at 200 nM each (E2aF 5′ ACC TAC TCC TGC CCC ACA GA 3′ and E2aR 5′ GTC CCC AGA AAT TGA CTG AGA GA 3′). In total, each set (Telomere and Eif2a) was run 4 separate times on 4 separate days. The average cycle time (Ct) for each was used to calculate the telomere to T/S ratio (T/S ratio = 2^-Ct(Telomere)-Ct(Eif2a)^ or 2^−ΔCt^). The relative T/S ratio was determined by dividing the T/S ratio of each participant by the average T/S ratio of all participants, and is expressed here as rLTL. Any samples that appeared as a potential outlier (>2 standard deviations from the mean) was repeated on a freshly prepared sample. The intra- assay percent coefficient of variation (% CV) were calculated for the quadruplicates. The average intra-assay % CV for the telomere ranged from 0.5–3.2% for all 96 participants and from 0.2–2.7% for the single copy gene for 95 participants. The one outlier had a% CV of 7.2% which was likely caused by a pipetting error in one of the 4 replicates creating a larger than average standard deviation. The inter-assay % CV was 1.8% for the telomere and 1.15% for the single copy gene. The correlation coefficient for telomere samples was between 0.84 and 0.93 in cross comparisons between the 4 replicates.

### 2.4. Potential Confounding Variables

Potential confounding sociodemographic, lifestyle and health-related variables were identified by the literature as being related to rLTL. The self-reported data were obtained via an online survey administered using the REDCap (Research Electronic Data Capture) survey software [32] hosted at the West Virginia Clinical and Translation Science Institute. Anthropometric measures, blood pressure and blood for analyses were obtained at an in-person health assessment visit.

#### 2.4.1. Sociodemographic and Lifestyle Factors

Sociodemographic (age, sex, income level, and education) and lifestyle factors (alcohol use, smoking history, physical activity, and sleep) were assessed using self-reported survey responses. For analysis, alcohol intake was categorized as “non-drinker”, “1–2 drinks per week” and “≥3 drinks per week”. Smoking history was classified as “having ever smoked” or “never having had smoked”; all participants were nonsmokers at the time of the study. Physical activity was estimated using the validated International Physical Activity Questionnaire (IPAQ) Short Form [33] and scoring protocol [34]. A total physical activity score (metabolic equivalent of task (MET)-minutes/week) was calculated. Activity was categorized as active, minimally active or inactive using the provided algorithm [34]. Sleep quality was evaluated using the validated 19-question Pittsburgh Sleep Quality Index (PSQI) [35]. Component scores were summed to obtain a global sleep quality score ranging from 0–21 points with higher scores indicating overall poorer sleep quality [35]. Global scores ≤ 5 were classified as “good” and > 5 as “poor” sleep [35]. For both the PSQI and IPAQ scoring, participant responses were excluded if any question was left blank or the answer was out-of-range [34,35].

#### 2.4.2. Health-Related Factors

Health-related factors included measures of adiposity, diagnoses of select health conditions, cardiovascular health (CVH) as determined by Life’s Simple 7 (LS7) and select inflammatory markers. Adiposity was assessed using body mass index (BMI), waist circumference (WC) and fat mass index (FMI). Anthropometric measures including height, weight, WC and body composition were taken at the in-person visit using standardized protocols with participants fasted, lightly clothed and without shoes. All measurements were recorded in duplicate and averages were used for analysis. Height (cm) was measured using the Seca 274 digital mobile stadiometer (Seca, Hamburg, Germany). Weight (kg) and FMI (kg/m^2^) were obtained using the Seca medical Bioelectrical Composition Analyzer (mBCA) 514 and Seca analytics 115 PC software (Seca, Hamburg, Germany) [36]. WC was measured using a Gulick II tape measure at the iliac crest. Values >102 cm for men and >88 cm for women were categorized as “at risk” [37]. BMI was calculated as weight (kg)/height (m^2^) and classified using World Health Organization classifications [37].

Participants were classified as having a diagnosis of pre-diabetes or diabetes, hypertension or dyslipidemia if they self-reported having been diagnosed with the health condition, they reported taking a medication used to treat the condition on the in-person questionnaire, or their measured lab values met standard diagnostic criteria. Each person’s CVH was assessed using the American Heart Association’s LS7 metric and scoring algorithm [38]. The LS7 metric assesses CVH based on 7 components: blood pressure, total cholesterol, blood glucose, physical activity, diet, BMI, and smoking status. Physical activity, diet information (except sodium intake), and smoking status were self-reported by participants. Sodium intake values were obtained from the 24-h recall data. Anthropometric measures for BMI, blood pressure and blood for analysis of total cholesterol and fasting blood glucose were collected by research personnel and a trained phlebotomist at an in-person health assessment visit. Total cholesterol and fasting blood glucose were measured by West Virginia University Medicine Hospital Labs. Blood pressure was measured by research personnel using the Omron HEM-907XL Intellisense^®^ Automatic Oscillatory Digital Blood Pressure automatic inflation sphygmomanometer (Omron Health Care, Lake Forest, IL, USA) using standard protocol. Each of the 7 factors was assigned a score of 0 (poor), 1 (intermediate) and 2 (ideal) based on AHA criteria [38]. Individual scores were summed for a total score ranging from 0–14, with higher scores corresponding to less risk of developing CVD. Total scores were categorized into a health risk group: poor (0–4), average (5–9), and optimum (10–14).

Three inflammatory markers associated with cardiovascular health were assessed: C-reactive protein (CRP), fibrinogen and TNF-α. Fibrinogen and C-reactive protein were measured by West Virginia University Medicine Hospital Labs. TNF-α levels were measured by Enzyme-Linked Immunosorbent Assay (ELISA) using 100 µL of sera and the Human TNF-α ELISA kit from Invitrogen (Product KHC3011) following the manufacture’s guidelines. The ELISAs were performed on 3 separated days and average values were calculated.

### 2.5. Statistical Analyses

The initial screening of explanatory variables including the diet factors and covariates was completed on all subjects (*n* = 96), but focused on a subset of the data (50%) with the most extreme values of telomere length, namely, the first (Q1) and fourth (Q4) quartile (*n* = 24 in each). Primary bivariate analyses included *t*-test, Chi-square test and Fisher’s Exact tests. *t*-tests were used to confirm differences in the rLTL between Q1 and Q4. The relationship of individual categorical variables (sociodemographic, lifestyle, health and diet factors) with Q1 and Q4 rLTL quartiles were examined using Chi-square test or Fisher’s Exact test when necessary due to the sample size restrictions. A logistic regression (LR) was used to measure the strength of the relationship of individual continuous variables to Q1 and Q4 of rLTL. The logit estimates of slope (β), standard error of the β (SE), as well as odds ratios with confidence limits and p-values corresponding to the slope estimates were reported for LR.

Continuous rLTL was used in a secondary set of analyses. All observations were included (*n* = 96) unless there were missing data or influential observations. Influence diagnostic tests were computed for the bivariate fit of continuous data using simple linear regression. Data point(s) which exceeded the criteria of student residual >2 (no more than 5 observations) were excluded in one round of screening. The relationship of each individual diet quality index scores as continuous variables and rLTL was first assessed by simple linear regression analysis (Model 1). Further, variables found significantly related to rLTL in the primary bivariate analyses (smoking, LS7, and income) together with the individual diet quality variables (HEI-2015, aMed and DST) were entered into multivariate models with an estimation method using residual maximum likelihood (REML), utilizing PROC MIXED of SAS^®^. Physical activity, although significant in the bivariate analysis, was not used in the secondary models due to a large reduction in sample size as many participants did not respond to all of the questions (*n* = 31, 32%). The multivariate models enabled us to assess if the specific dietary patterns would be related to rLTL when adjusted for other significant characteristics, such as LS7 and smoking (Model 2), with further examination of the diet and smoking interaction (Model 3) and additional adjustments for income (Model 4). The outcome of such an analysis included the F-value, determined from the ratio of explained and unexplained variability for the main effect and interaction, and the corresponding p-value in addition to specific parameter estimates, such as the slope (β), its standard error (SE), the *t*-value, a test-statistics for testing β = 0, and a corresponding *p*-value for each continuous variable and for c-1 categories for categorical variables. For smoking, the β for the effect of the “never smoked” group was estimated; for income, the <$50,000 and between $50,000 and $75,000 groups were estimated; for DST, the categorical “at nutrition risk” group effect on rLTL was estimated, while the remaining last category in each variable could not be assessed due to the loss of degrees of freedom. The quality of each model was estimated by Akaike Information Criterion with a sample size correction (AICC) [39,40]. The AICC approximates the quality of each model and is determined from a restricted maximum log-likelihood, the number of observations, and the number of parameters in the model; the smallest AICC represents an optimal model.

Data were analyzed using JMP and SAS software (JMP^®^, Version Pro 12.2, SAS Institute Inc., Cary, NC, USA, Copyright ©2015; SAS^®^, Version 9.4, SAS Institute Inc., Cary, NC, USA, Copyright ©2002–2012). The significance criterion alpha for all tests was 0.05.

## 3. Results

### 3.1. Sociodemographic, Lifestyle and Health-related Characteristics

Ninety-six participants took part in the study (57.3% women). Overall, participants were predominately white (97%), were college educated (57.3%) and had an annual household income of $50,000 or more (67%). On average, participants were 54.3 years old and had a BMI of 30.9 kg/m^2^ (SD = 7.2). Half of the participants (51%) were obese, and a large majority had dyslipidemia (91.7%) and an elevated waist circumference (72.9%). All participants had at least one risk factor for CVD.

The rLTL values ranged from 0.46 to 1.49 (mean ± SD was 1.02 ± 0.18). Participant characteristics for the shortest and longest quartiles of rLTL are provided in Table 1. Of the sociodemographic variables, rLTL was only associated with income. Participants with a yearly income at or above $75,000 had significantly longer telomeres than participants with an annual income below $50,000. Of those in the highest rLTL quartile, 65.2% had incomes ≥$75,000 compared to 29.2% in the lowest quartile (*p* = 0.02).

Of the lifestyle factors, physical activity and smoking history, but not alcohol consumption and sleep, were related to rLTL. A significant relationship was found between rLTL and physical activity (*p* = 0.02) and smoking history (*p* = 0.01). None of the participants were current smokers as this was an exclusion factor for study participation. However, half of participants had smoked in the past with a majority of previous smokers (72.9%) having quit more than 5 years prior to the study. Of those in the highest rLTL quartile, 83.3% had never smoked compared to 41.2% in the lowest quartile (*p* = 0.01).

Of the health-related factors, only cardiovascular health as measured by the LS7 on both a continuous and categorical scale was related to rLTL quartiles. About half (52.2%) were rated on the LS7 as having “average” cardiovascular health, 47.8% rated as “optimal” and none were identified as having “poor” cardiovascular health. Those in the highest rLTL quartile had higher total scores on the LS7 survey than those in the lowest quartile (9.22 ± 1.81 vs. 7.76 ± 1.73, *p* = 0.01). Of the participants in the highest quartile of rLTL, 52% had optimal cardiovascular health compared to only 19.1% of participants in the lowest quartile (*p* = 0.02).

**Figure 1:**
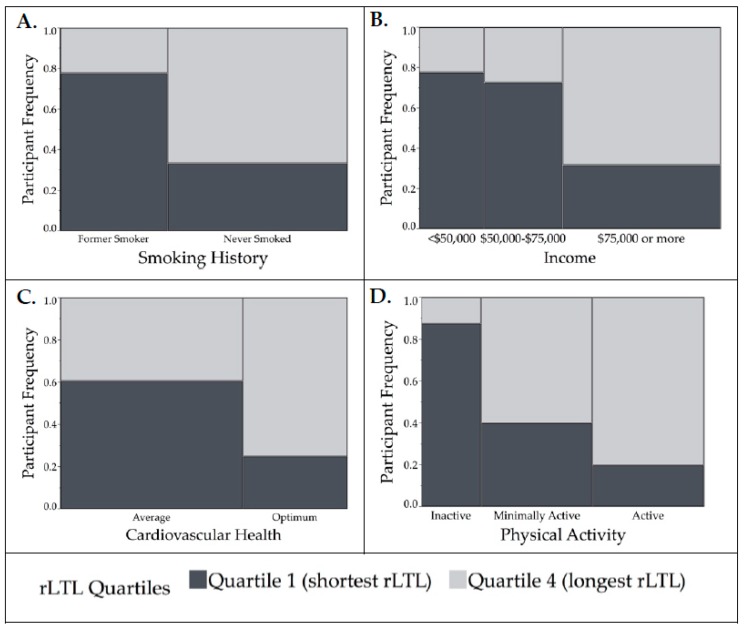
Mosaic plots demonstrating the relationship between significant covariates and relative leukocyte telomere length (rLTL) (Quartile 1 (shortest rLTL) and Quartile 4 (longest rLTL)) analyzed using Fisher’s exact test. Height of bars represents proportion of participants in each category and their quartile of rLTL, the width of each bar is proportional to sample size. (**A**) For smoking history (*n* = 48), more participants that had never smoked had longer rLTL compared to former smokers (*p* = 0.01). (**B**) For income (*n* = 42), participants that had earned ≥$75,000 had the longest rLTL (*p* = 0.02). (**C**) Cardiovascular health measured by Life’s Simple 7 metric (*n* = 44) was associated with rLTL; more participants with “optimal” cardiovascular health had longer rLTL than those with “average” cardiovascular health (*p* = 0.02). (**D**) Physical activity was measured using the International Physical Activity Questionnaire (*n* = 38). More participants that were active and had longer rLTL than those who were inactive (*p* = 0.01).

Overall, based on the quartiles analyses, the smoking history, cardiovascular health (LS7 scores), income, and physical activity differed significantly between the lowest and highest rLTL quartiles and thus were considered as potential confounding variables (Figure 1). However, physical activity was not included in further models as data was missing for nearly 1/3 of the sample (*n* = 31).

### 3.2. Diet Quality and Nutrition Risk

Association of nutrition risk categories with rLTL quartiles (shortest and longest) were examined by Chi-square test. Relationship of continuous diet quality scores with rLTL quartiles (shortest and longest) were assessed by logistic regression and results of both analyses are listed in Table 2. A majority of participants (64.6%) were classified as “at nutrition risk” using the DST as a categorical measure. An association of nutrition risk and rLTL category was detected (*p* = 0.02); a large proportion of participants with shorter rLTL were at nutrition risk (79.2%) while only 45.8% of those with the longest rLTL were at nutrition risk. Using LR, no effect of diet quality scores (aMed, HEI-2015, and DST measured on the continuous scale) on the shortest and longest quartiles of rLTL were observed as demonstrated by odds ratios values close to one. That is, the odds of having higher rLTL (Q4) did not increase with a higher diet quality scores with respect to Q1.

Table 3 lists the associations between diet quality scores (aMed, HEI-2015, and DST) and telomere length (continuous) examined individually (Model 1) or after adjustment for LS7 and smoking (Model 2), diet quality, and smoking interaction (Model 3) and with the addition of the income category (Model 4). Categorical DST (<60 “at nutrition risk” and ≥60 “potential risk/ not at risk”) was found significantly associated with rLTL in the unadjusted model (Model 1). The least square mean (LSM) telomere length ± standard error (SE) was 0.98 ± 0.02 for the “at nutrition risk” group and 1.07 ± 0.03 for the “potential/no risk group”. In addition, the effect of the DST category on rLTL was observed in the model with an adjustment for smoking, LS7, and income (Model 4, *p* = 0.02). None of the continuous diet quality scores were related to rLTL when used in a simple model (Model 1) without any covariates. However, a trend approaching statistical significance (*p* = 0.07) was demonstrated in the overall F-value of aMed in Model 3, when the effect of aMed on rLTL was adjusted to smoking plus the interaction of aMed and smoking (not shown). Smoking history was significantly related to rLTL in many models tested (Table 3). Individuals who never smoked had a longer telomere, 1.08 (LSM) ± 0.02 (SE), compared to those who ever smoked, 0.98 (LSM) ± 0.02 (SE).

**Figure 2:**
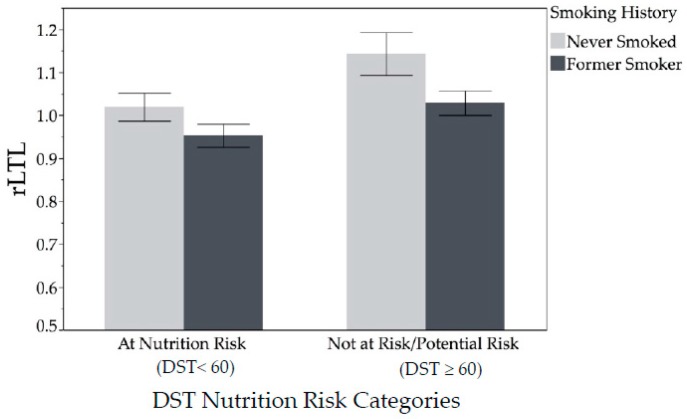
Relative leukocyte telomere length (rLTL) by smoking history and nutrition risk category (*n* = 92) analyzed using multivariate modeling. Participants who had never smoked had higher rLTL than those who were former smokers across both categories of nutrition risk. Likewise, those who are not at nutrition risk had higher rLTL across both categories of smoking. Those who had never smoked and were not at nutrition risk had the longest rLTL, although there was no significant interaction effect detected. Abbreviation: DST, Dietary Screening Tool.

Graphical representation of significant relationship of rLTL with smoking history and nutrition risk category is in Figure 2. Participants who had never smoked had longer rLTL than those who smoked in their past across both categories of nutrition risk (main effect of Smoking, F-Value = 11.82, *p* = 0.0009). Likewise, participants who were at nutrition risk, regardless of smoking in the past, had lower rLTL (main effect of DST, F-value = 8.95, *p* = 0.004). However, no significant interaction was detected. Figure 2 shows rLTL by smoking history and nutrition risk category. Participants who had never smoked had longer rLTL than those who smoked in their past, across both categories of nutrition risk. Likewise, participants who were at nutrition risk, regardless of smoking in the past, had lower rLTL. However, no significant interaction was detected.

## 4. Discussion

To our knowledge, this is the first study to determine the relationships between evidence-based dietary patterns and nutrition risk classifications with rLTL in a subpopulation at risk for CVD. In this cross-sectional study of 96 middle-aged adults with at least one risk factor for CVD, participants identified as being “at nutrition risk” (DST scores < 60) had shorter rLTL than those “not at risk/potential risk” (DST scores > 60). However, we did not detect associations between rLTL and any of the diet quality scores (HEI-2015, aMed, or DST) measured on a continuous scale.

The discrepancy between the results for DST categorical versus continuous data may be in the robust nature of categorical estimates (Chi-Square test) as opposed to the more refined nature of linear regression analysis. That is, the rate of change in rLTR due to one-point increase in DST 100-point scale may be negligible, while the logical grouping may elude to the threshold of nutritional quality and risk status. Nutrition risk was determined based on scores from the DST. In the DST validation study, those classified “at nutrition risk” had lower intakes of several antioxidant nutrients, and lower serum levels of carotenoids than those “not at risk” [30]. Thus, a plausible mechanism for the relationship between DST risk categories and rLTL is that lower intakes of antioxidant nutrients result in higher oxidative stress levels [41], a major cause of DNA damage, which exacerbates telomere shortening [42]. Additionally, higher serum carotenoids have been associated with longer LTL [43,44].

We did not detect a relationship between either of the diet index scores and rLTL, however, we noticed a value approaching significance for the energy-adjusted aMed (*p* = 0.07) in a model with adjustment to smoking and aMed and smoking interaction. Several other studies have been conducted on a Mediterranean style diet and TL, but they use various diet indices and statistical models; thus, results are inconsistent. A study on older adults did not detect an association between diet and LTL [45], another found a significant correlation in whites only [46], and a few [13,20,47] found significant positive associations. Unlike the other indices, the aMed diet index score is measured in relation to the median intake of the sample. The only other study to use this specific index found a significant relationship between diet and rLTL in middle-aged and older women [20]; however, they had a very large sample size and healthy subjects. A subsample of the PREDIMED trial of middle-aged and older adults at risk for CVD, found that baseline adherence to a Mediterranean diet assessed using a 14-item tool was related to TL in women, but not men [48]. In our study, the mean aMed score was low in both the shortest and longest quartiles (3.63 ± 1.71 and 4.04 ± 2.16, respectively) representing an overall low to moderate adherence to Mediterranean-style diet. While no other studies were found that used the updated HEI-2015, Leung et al. used the HEI-2010 in a large population-based sample and found a significant difference in TL between the highest and lowest quintiles of diet quality in healthy women, but not men [20].

Our findings that rLTL correlated with smoking status, physical activity and income were consistent with the literature [49,50,51]. Interestingly, our population was made up of non-smokers, but having had smoked in the past was related to rLTL in most models. A systematic review and meta-analysis concluded that those who never smoked had longer telomeres than those who had ever smoked [52]. We did not detect a significant difference in rLTL based on sex, age, BMI, or other CVD risk factors. Several studies indicate that women have longer TL than men; however, a systematic review and meta-analysis concluded that the difference was significant in studies that measured TL using Southern blot method, but not PCR as we did here [53]. We may not have detected a difference in age as other studies have because our population included only middle-aged adults. Most studies designed to assess the effects of chronological age on rLTL also include younger and older adults in the sample.

We did, however, detect a relationship between rLTL and LS7 scores which are made up of seven different CVD risk factors. This supports the notion that the combined effect of multiple risk factors has a greater impact on rLTL than individual risk factors. This finding was confirmed by Gebreab et al. in a large population based study on the association between CVH and LTL using NHANES data [7]. Their findings showed that those with “ideal” CVH as measured by LS7 had longer LTL than those with “intermediate” and “poor” CVH [7]. Thus, achieving the AHA’s ideal CVH may slow LTL shortening rate, particularly in non-Hispanic whites. The components of LS7 are potentially modifiable, thus intervention studies are needed to determine if lifestyle improvements resulting in higher LS7 scores (i.e., better cardiovascular health) result in reduced telomere attrition over time, particularly in subpopulations at risk for CVD.

The strengths of the present study include the collection of multiple 24-h dietary recalls by trained research personnel; a detailed lifestyle assessment using validated survey instruments; and objective measures of blood pressure, anthropometrics and laboratory values. There are some limitations to note. First, we had a small sample size composed predominantly of non-Hispanic whites. Therefore, our findings may not be generalized to other populations or ethnicities. Secondly, the diet, lifestyle and health assessments were all based on self-reported data which is prone to recall and other biases, despite being validated measurement tools that are routinely used to assess lifestyle factors [54]. Thirdly, the LTL was analyzed using DNA isolated from peripheral blood mononuclear cells (PBMC) [31] and this may not necessarily reflect TL in other cells and tissue throughout the body. However, the measures generally correlate across tissues within the same individual [13]. Lastly, the cross-sectional design implies association rather than causal relationships limiting the clinical interpretations of the findings. Longitudinal and interventional studies that measure rLTL over time are needed to better understand the relationship of diet to rLTL.

## 5. Conclusions

We found evidence that individuals with a higher quality diet as assessed by the DST had longer telomeres than those with lower quality diets. Participants identified as being “at nutrition risk” by the DST had shorter rLTL than those “not at risk/potential risk”. However, adherence to the DGAs or a Mediterranean diet was not associated with rLTL in unadjusted models or models adjusted for confounding variables. Intervention studies are needed to determine if improving diet quality of those at nutrition risk result in telomere attrition or lengthening over time.

## Figures and Tables

**Table 1:** The sociodemographic, lifestyle and health-related factors by shortest and longest quartiles of relative leukocyte telomere length (rLTL).

	Total	Q1 (Shortest rLTL)	Q4 (Longest rLTL)	*p*-value ^1^
rLTL, mean ± SD	1.02 ± 0.18	0.81 ± 0.09	1.25 ± 0.10	
rLTL, range	0.46–1.49	0.46–0.91	1.13–1.49	<0.0001
**Sociodemographic Factors**				
Sex, women	55 (57.3%)	13 (54.2%)	16 (66.7%)	0.38
Yearly income				0.02
<$50,000	22 (23.2%)	7 (29.2%)	2 (8.7%)	
$50,000–$74,999	20 (21.0%)	8 (33.3%)	3 (13.1%)	
≥$75,000	44 (46.3%)	7 (29.2%)	15 (65.2%)	
No response	9 (9.5%)	2 (8.3%)	3 (13.0%)	
**Education**				0.46
Grade 9–12 or GED	17 (17.7%)	4 (16.7%)	5 (20.8%)	
College 1–3 years	24 (25.0%)	8 (33.3%)	4 (16.7%)	
College ≥ 4 years	55 (57.3%)	12 (50.0%)	15 (62.5%)	
**Lifestyle Factors**				
Smoking history, never smoked	48 (50.0%)	10 (41.2%)	20 (83.3%)	0.01
Alcohol Consumption				0.32
0 drinks/week	33 (34.4%)	11 (45.8%)	7 (29.2%)	
1–2 drinks/week	38 (39.6%)	7 (29.2%)	12 (50.0%)	
≥3 drinks/week	25 (26.0%)	6 (25.0%)	5 (20.8%)	
Physical Activity Category ^2^				0.01
Active	22 (31.4%)	3 (18.8%)	12 (54.6%)	
Minimally active	29 (41.4%)	6 (37.5%)	9 (40.9%)	
Inactive	19 (27.1%)	7 (43.8%)	1 (4.6%)	
Sleep quality Category ^3^				0.30
Poor sleep quality (>5)	48 (52.2%)	14 (60.9%)	10 (45.5%)	
Good sleep quality (≤5)	44 (47.8%)	9 (39.1%)	12 (54.5%)	
**Health Related Factors**				
Elevated waist circumference ^4^	70 (72.9%)	20 (83.3%)	15 (62.5%)	0.10
BMI Category				0.22
Normal (18.5–24.9 kg/m^2^)	22 (22.9%)	4 (16.7%)	7 (29.2%)	
Overweight (25.0–29.9 kg/m^2^)	25 (26.0%)	6 (25.0%)	9 (37.5%)	
Obese (≥30 kg/m^2^)	49 (51.1%)	14 (58.3%)	8 (33.3%)	
Diabetes/prediabetes	34 (35.4%)	11 (45.8%)	7 (29.2%)	0.38
Hypertension	37 (38.5%)	9 (37.5%)	9 (37.5%)	1.00
Dyslipidemia	88 (91.7%)	23 (95.8%)	21 (87.5%)	0.30
“Average” cardiovascular health ^5^	48 (52.2%)	17 (80.9%)	11 (48%)	0.02
**Continuous Factors (Q1 = reference)**	**β (SE)**	**Odds Ratio (95% Wald Confidence Limits)**		***p*-value**
Age, year	0.1011 (0.066)	1.106 (0.97–1.26)		0.12
C-reactive protein (CRP), mg/L	0.000625 (0.029)	1.001 (0.95–1.06)		0.98
Fibrinogen, mg/dL	0.00101 (0.003)	1.001 (0.99–1.01)		0.76
TNF-alpha, pg/mL	0.00102 (0.08)	1.001 (0.86–1.17)		0.99

The values of rLTL are mean ± SDs or *n* (%). *n* = 96 except for income and marital status (*n* = 95), physical activity (*n* = 71), and Life’s Simple 7 (*n* = 90). Abbreviations: rLTL = relative Leukocyte Telomere Length; BMI = Body Mass Index. The rLTL values between Q1 and Q4 were compared by *t*-test. ^1^ Associations of categorical variables to shortest (Q1) and longest (Q4) quartiles of rLTL were examined using Chi-square test or Fisher’s Exact test. Relationship of continuous explanatory factors to Q1 and Q4 categories of rLTL were examined by logistic regression and the logit estimates of slope (β), standard error of the β (SE), as well as odds ratios with confidence limits and p-values corresponding to the slope estimates are listed. ^2^ International Physical Activity Questionnaire ^3^ Pittsburgh Sleep Quality Index; scores range from 0–21. ^4^ Elevated waist circumference: ≥102 cm in men or ≥88 cm in women. ^5^ LS7 categories included: average cardiovascular health (scores 5–9) and optimum (10–14).

**Table 2:** Cross classification of diet quality scores by relative leukocyte telomere length (rLTL).

	All	Q1 (Shorter rLTL)	Q4 (Longer rLTL)	
	*n* = 96	*n* = 24	*n* = 24	
**rLTL**				
rLTL, mean ± SD	1.02 ± 0.18	0.81 ± 0.09	1.25 ± 0.10	
rLTL, range	0.46–1.49	0.46–0.91	1.13–1.49	
**Nutrition Risk (categorical)**				***p*-value** ^1^
At risk (DST < 60)	62 (64.6%)	19 (79.2%)	11 (45.8%)	0.02
Not at-risk/potential risk (DST ≥ 60)	34 (35.4%)	5 (20.8%)	13 (54.2%)	
**Diet Quality Scores (continuous)**	**β (SE)**	**Odds Ratio (95% Wald Confidence Limits)**		***p*-value** ^2^
aMed (0–9) (Q1 = reference)	0.1141 (0.15)	1.121 (0.82–1.51)		0.45
HEI-2015 (0–100) (Q1 = reference)	0.0254 (0.02)	1.026 (0.983–1.07)		0.24
DST (0–100) (Q1 = reference)	0.064 (0.024)	1.037 (0.99–1.09)		0.12

Values of rLTL are mean ± SD or *n* (%). Abbreviations: aMed, alternate Mediterranean Diet (energy adjusted); HEI, Healthy Eating Index 2015; DST, Dietary Screening Tool. ^1^ Relationship of nutritional risk (categorical DST) and rLTL category examined using Chi-square test. ^2^ The relationship between continuous diet quality scores and the extreme rLTL (Q1 and Q4) were estimated by logistic regression. Logit estimates of slope (β), standard error of the β (SE), as well as odds ratios with confidence limits and corresponding *p*-values are reported.

**Table 3:** Relationships of diet quality index scores (explanatory variables) and relative leukocyte telomere length (continuous response variable) in unadjusted models and after adjusting for confounding factors ^1^.

Explanatory Variable		*n*	β (SE) ^2^	*t*-value ^3^	*p*-value ^4^	AICC ^5^
**aMed (energy adjusted, Continuous)**						
Model 1	aMed	94	0.008 (0.01)	0.87	0.39	−57.6
Model 2	aMed	88	0.006 (0.01)	0.54	0.59	−55.8
	LS7		0.014 (0.01)	1.54	0.13	
	Smoking (never smoked)		0.081 (0.04)	2.26	0.03	
Model 3	aMed	94	0.012 (0.01)	0.89	0.37	−58.4
	Smoking (never smoked)		0.077 (0.08)	0.98	0.33	
	aMed x Smoking (never smoked)		0.011 (0.02)	0.56	0.57	
Model 4	aMed	79	0.010 (0.01)	0.92	0.36	−38.8
	LS7		0.005 (0.01)	0.52	0.60	
	Smoking (never smoked)		0.083 (0.04)	2.19	0.03	
	Income (<50 K/year)		−0.046 (0.04)	−1.04	0.30	
	Income (50–75 K/year)		−0.039 (0.04)	−0.86	0.40	
**Healthy Eating Index–2015 (Continuous)**						
Model 1	HEI-2015	91	0.001 (0.001)	0.88	0.38	−65.4
Model 2	HEI-2015	85	−0.0004 (0.001)	−0.35	0.73	−65.5
	LS7		0.018 (0.01)	2.22	0.03	
	Smoking (never smoked)		0.062 (0.03)	1.98	0.05	
Model 3	HEI-2015	91	0.0003 (0.001)	0.23	0.82	−61.1
	Smoking (never smoked)		−0.056 (0.13)	−0.45	0.66	
	HEI-2015 × smoking		0.003 (0.002)	1.27	0.21	
Model 4	HEI-2015	76	0.0003 (0.001)	0.23	0.82	−48.2
	LS7		0.009 (0.009)	0.97	0.33	
	Smoking (never smoked)		0.061 (0.03)	1.84	0.07	
	Income (<50 K/year)		−0.066 (0.04)	−1.39	0.10	
	Income (50–75 K/year)		−0.057 (0.04)	1.81	0.17	
**Dietary Screening Tool (Continuous)**						
Model 1	DST	92	0.002 (0.001)	1.35	0.18	−60.7
Model 2	DST	86	0.00008 (0.001)	0.06	0.95	−59.3
	LS7		0.016 (0.008)	1.84	0.07	
	Smoking (never smoked)		0.074 (0.03)	2.36	0.02	
Model 3	DST	92	0.003 (0.002)	1.51	0.14	−55.9
	Smoking (never smoked)		0.196 (0.14)	1.4	0.16	
	DST × smoking		−0.002 (0.003)	−0.7	0.49	
Model 4	DST	77	0.003 (0.001)	0.22	0.83	−41.2
	LS7		0.009 (0.01)	0.92	0.36	
	Smoking (never smoked)		0.072 (0.03)	2.14	0.04	
	Income (<50 K/year)		−0.043 (0.04)	−1.04	0.30	
	Income (50–75 K/year)		−0.057 (0.04)	−1.32	0.19	
**Dietary Screening Tool (Categorical)**						
Model 1	DST Category (At risk, <60)	92	−0.091 (0.03)	−2.76	0.007	−74.4
Model 2	DST Category (At risk, <60)	86	−0.059 (0.03)	3.07	0.08	−70.6
	LS7		0.011 (0.01)	1.35	0.18	
	Smoking (never smoked)		0.079 (0.03)	2.58	0.01	
Model 3	DST Category (At risk, <60)	92	−0.068 (0.04)	−1.55	0.12	−76.8
	Smoking (never smoked)		0.133 (0.05)	2.65	0.01	
	DST Category × smoking		−0.051 (0.06)	−0.81	0.42	
Model 4	DST Category (At risk, <60)	77	−0.088 (0.04)	−2.49	0.02	−57.6
	LS7		−0.001 (0.01)	−0.11	0.92	
	Smoking (never smoked)		0.072 (0.03)	2.28	0.03	
	Income (<50 K/year)		−0.093 (0.04)	−2.34	0.02	
	Income (50–75 K/year)		−0.067 (0.04)	−1.67	0.1	

^1^ Models of fitting rLTL (continuous) from unadjusted diet quality scores (Model 1, simple linear regression), after adjustment to LS7 and smoking history (Model 2), after adjustment to interaction of diet quality and smoking (Model 3), and after additional adjustment for income (Model 4). Models 2-4 are multivariate covariance models with combination of continuous (aMed, HEI-2015, DST, LS7) and categorical (smoking and Income) explanatory variables using PROC MIXED of SAS^Ò^. ^2^ Parameter estimates (β = slope) and standard error (SE) of the estimate of the factor in the model. ^3^ *t*-value tests the null hypothesis β = 0 meaning that the parameter (slope) is zero, thus that model component does not affect the rLTL. ^4^ Significant *p*-value <0.05. ^5^ AICC, Akaike Information Criterion with Correction for small sample size is a criterion of goodness of fit of the model; smallest AICC is considered an optimal model. The overall optimal model (AICC = −76.8) is based on categorical DST with adjustment to smoking in addition to DST and smoking interaction. Abbreviations: aMed, alternate Mediterranean Diet; LS7, Life’s Simple 7; HEI-2015, Healthy Eating Index 2015; DST, Dietary Screening Tool.

## References

[1] Sanders J.L., Newman A.B. (2013). Telomere length in epidemiology: A biomarker of aging, age-related disease, both, or neither?. Epidemiol. Rev..

[2] Demissie S., Levy D., Benjamin E.J., Cupples L.A., Gardner J.P., Herbert A., Kimura M., Larson M.G., Meigs J.B., Keaney J.F. (2006). Insulin resistance, oxidative stress, hypertension, and leukocyte telomere length in men from the Framingham Heart Study. Aging Cell.

[3] Reichert S., Stier A. (2017). Does oxidative stress shorten telomeres in vivo? A review. Biol. Lett..

[4] Blackburn E.H., Epel E.S., Lin J. (2015). Human telomere biology: A contributory and interactive factor in aging, disease risks, and protection. Science.

[5] Childs B.G., Durik M., Baker D.J., van Deursen J.M. (2015). Cellular senescence in aging and age-related disease: From mechanisms to therapy. Nat. Med..

[6] Peng H., Mete M., Desale S., Fretts A.M., Cole S.A., Best L.G., Lin J., Blackburn E., Lee E.T., Howard B.V. (2017). Leukocyte telomere length and ideal cardiovascular health in American Indians: The Strong Heart Family Study. Eur. J. Epidemiol..

[7] Gebreab S.Y., Manna Z.G., Khan R.J., Riestra P., Xu R., Davis S.K. (2017). Less Than Ideal Cardiovascular Health Is Associated with Shorter Leukocyte Telomere Length: The National Health and Nutrition Examination Surveys, 1999–2002. J. Am. Heart Assoc..

[8] Mundstock E., Sarria E.E., Zatti H., Mattos Louzada F., Kich Grun L., Herbert Jones M., Guma F.T.C.R., Mazzola In Memoriam J., Epifanio M., Stein R.T. (2015). Effect of obesity on telomere length: Systematic review and meta-analysis. Obesity.

[9] Gardner J.P., Li S., Srinivasan S.R., Chen W., Kimura M., Lu X., Berenson G.S., Aviv A. (2005). Rise in insulin resistance is associated with escalated telomere attrition. Circulation.

[10] Tamura Y., Takubo K., Aida J., Araki A., Ito H. (2016). Telomere attrition and diabetes mellitus. Geriatr. Gerontol. Int..

[11] Bär C., Blasco M.A. (2016). Telomeres and telomerase as therapeutic targets to prevent and treat age-related diseases. F1000Research.

[12] Slagboom P.E., Droog S., Boomsma D.I. (1994). Genetic determination of telomere size in humans: A twin study of three age groups. Am. J. Hum. Genet..

[13] Crous-Bou M., Fung T.T., Prescott J., Julin B., Du M., Sun Q., Rexrode K.M., Hu F.B., De Vivo I. (2014). Mediterranean diet and telomere length in Nurses’ Health Study: Population based cohort study. BMJ.

[14] Tucker L.A. (2018). Dietary Fiber and Telomere Length in 5674 U.S. Adults: An NHANES Study of Biological Aging. Nutrients.

[15] Tucker L.A. (2017). Consumption of Nuts and Seeds and Telomere Length in 5582 Men and Women of the National Health and Nutrition Examination Survey (NHANES). J. Nutr. Health Aging.

[16] Rafie N., Golpour Hamedani S., Barak F., Safavi S.M., Miraghajani M. (2017). Dietary patterns, food groups and telomere length: A systematic review of current studies. Eur. J. Clin. Nutr..

[17] Song Y., You N.-C.Y., Song Y., Kang M.K., Hou L., Wallace R., Eaton C.B., Tinker L.F., Liu S. (2013). Intake of small-to-medium-chain saturated fatty acids is associated with peripheral leukocyte telomere length in postmenopausal women. J. Nutr..

[18] Leung C.W., Laraia B.A., Needham B.L., Rehkopf D.H., Adler N.E., Lin J., Blackburn E.H., Epel E.S. (2014). Soda and cell aging: Associations between sugar-sweetened beverage consumption and leukocyte telomere length in healthy adults from the National Health and Nutrition Examination Surveys. Am. J. Public Health.

[19] Fretts A.M., Howard B.V., Siscovick D.S., Best L.G., Beresford S.A., Mete M., Eilat-Adar S., Sotoodehnia N., Zhao J. (2016). Processed Meat, But Not Unprocessed Red Meat, Is Inversely Associated with Leukocyte Telomere Length in the Strong Heart Family Study. J. Nutr..

[20] Leung C.W., Fung T.T., McEvoy C.T., Lin J., Epel E.S. (2018). Diet Quality Indices and Leukocyte Telomere Length among Healthy US Adults: Data from the National Health and Nutrition Examination Surveys (NHANES) 1999–2002. Am. J. Epidemiol..

[21] Reedy J., Krebs-Smith S.M., Miller P.E., Liese A.D., Kahle L.L., Park Y., Subar A.F. (2014). Higher diet quality is associated with decreased risk of all-cause, cardiovascular disease, and cancer mortality among older adults. J. Nutr..

[22] Segal L.M., Rayburn J., Beck S.E. (2017). The State of Obesity: Better Policies for a Healthier America 2017.

[23] Lee-Kwan S.H., Moore L.V., Blanck H.M., Harris D.M., Galuska D. (2017). Disparities in State-Specific Adult Fruit and Vegetable Consumption—United States, 2015. MMWR Morb. Mortal. Wkly. Rep..

[24] Nutrition Coordination Center (NCC), University of Minnesota Guide to Creating Variables, Needed to Calculate Scores for Each Component of the Healthy Eating Index-2015 (HEI-2015). http://www.ncc.umn.edu/ndsrsupport/hei2015.pdf.

[25] National Cancer Institute Division of Cancer Control and Population Sciences The Healthy Eating Index Research Uses: Overview of the Methods & Calculations. https://epi.grants.cancer.gov/hei/hei-methods-and-calculations.html.

[26] Developing the Healthy Eating Index. https://epi.grants.cancer.gov/hei/developing.html.

[27] Fung T.T., Rexrode K.M., Mantzoros C.S., Manson J.E., Willett W.C., Hu F.B. (2009). Mediterranean diet and incidence of and mortality from coronary heart disease and stroke in women. Circulation.

[28] Fung T.T., McCullough M.L., Newby P.K., Manson J.E., Meigs J.B., Rifai N., Willett W.C., Hu F.B. (2005). Diet-quality scores and plasma concentrations of markers of inflammation and endothelial dysfunction. Am. J. Clin. Nutr..

[29] Bailey R.L., Miller P.E., Mitchell D.C., Hartman T.J., Lawrence F.R., Sempos C.T., Smiciklas-Wright H. (2009). Dietary screening tool identifies nutritional risk in older adults123. Am. J. Clin. Nutr..

[30] Ventura Marra M., Thuppal S.V., Johnson E.J., Bailey R.L. (2018). Validation of a Dietary Screening Tool in a Middle-Aged Appalachian Population. Nutrients.

[31] Cawthon R.M. (2002). Telomere measurement by quantitative PCR. Nucleic Acids Res..

[32] Harris P.A., Taylor R., Thielke R., Payne J., Gonzalez N., Conde J.G. (2009). Research electronic data capture (REDCap)—A metadata-driven methodology and workflow process for providing translational research informatics support. J. Biomed. Inform..

[33] Craig C.L., Marshall A.L., Sjöström M., Bauman A.E., Booth M.L., Ainsworth B.E., Pratt M., Ekelund U., Yngve A., Sallis J.F. (2003). International physical activity questionnaire: 12-country reliability and validity. Med. Sci. Sports Exerc..

[34] International Physical Activity Questionnaire IPAQ (2005) Guidelines for Data Processing and Analysis of the International Physical Activity Questionnaire (IPAQ)—Short and Long Forms, Revised on November 2005. https://sites.google.com/site/theipaq/scoring-protocol.

[35] Buysse D.J., Reynolds C.F., Monk T.H., Berman S.R., Kupfer D.J. (1989). The Pittsburgh Sleep Quality Index: A new instrument for psychiatric practice and research. Psychiatry Res..

[36] Bosy-Westphal A., Jensen B., Braun W., Pourhassan M., Gallagher D., Müller M.J. (2017). Quantification of whole-body and segmental skeletal muscle mass using phase-sensitive 8-electrode medical bioelectrical impedance devices. Eur. J. Clin. Nutr..

[37] National Heart, Lung, and Blood Institute Classification of Overweight and Obesity by BMI, Waist Circumference, and Associated Disease Risks. https://www.nhlbi.nih.gov/health/educational/lose_wt/BMI/bmi_dis.htm.

[38] Rebholz C.M., Anderson C.A.M., Grams M.E., Bazzano L.A., Crews D.C., Chang A.R., Coresh J., Appel L.J. (2016). Relationship of the American Heart Association’s Impact Goals (Life’s Simple 7) with Risk of Chronic Kidney Disease: Results from the Atherosclerosis Risk in Communities (ARIC) Cohort Study. J. Am. Heart Assoc. Cardiovasc. Cerebrovasc. Dis..

[39] Akaike H. (1974). A new look at the statistical model identification. IEEE Trans. Autom. Control.

[40] Hurvich C.M., Tsai C.-L. (1989). Regression and time series model selection in small samples. Biometrika.

[41] Åsgård R., Rytter E., Basu S., Abramsson-Zetterberg L., Möller L., Vessby B. (2007). High intake of fruit and vegetables is related to low oxidative stress and inflammation in a group of patients with Type 2 Diabetes. Scand. J. Food Nutr..

[42] D’Adda Di Fagagna F., Reaper P.M., Clay-Farrace L., Fiegler H., Carr P., Von Zglinicki T., Saretzki G., Carter N.P., Jackson S.P. (2003). A DNA damage checkpoint response in telomere-initiated senescence. Nature.

[43] Sen A., Marsche G., Freudenberger P., Schallert M., Toeglhofer A.M., Nagl C., Schmidt R., Launer L.J., Schmidt H. (2014). Association Between Higher Plasma Lutein, Zeaxanthin, and Vitamin C Concentrations and Longer Telomere Length: Results of the Austrian Stroke Prevention Study. J. Am. Geriatr. Soc..

[44] Nomura S.J., Robien K., Zota A.R. (2017). Serum Folate, Vitamin B-12, Vitamin A, γ-Tocopherol, α-Tocopherol, and Carotenoids Do Not Modify Associations between Cadmium Exposure and Leukocyte Telomere Length in the General US Adult Population. J. Nutr..

[45] Milte C.M., Russell A.P., Ball K., Crawford D., Salmon J., McNaughton S.A. (2018). Diet quality and telomere length in older Australian men and women. Eur. J. Nutr..

[46] Gu Y., Honig L.S., Schupf N., Lee J.H., Luchsinger J.A., Stern Y., Scarmeas N. (2015). Mediterranean diet and leukocyte telomere length in a multi-ethnic elderly population. AGE.

[47] Boccardi V., Esposito A., Rizzo M.R., Marfella R., Barbieri M., Paolisso G. (2013). Mediterranean Diet, Telomere Maintenance and Health Status among Elderly. PLoS ONE.

[48] García-Calzón S., Martínez-González M.A., Razquin C., Arós F., Lapetra J., Martínez J.A., Zalba G., Marti A. (2016). Mediterranean diet and telomere length in high cardiovascular risk subjects from the PREDIMED-NAVARRA study. Clin. Nutr..

[49] Tucker L.A. (2017). Physical activity and telomere length in U.S. men and women: An NHANES investigation. Prev. Med..

[50] Mirabello L., Huang W.-Y., Wong J.Y.Y., Chatterjee N., Reding D., Crawford E.D., De Vivo I., Hayes R.B., Savage S.A. (2009). The association between leukocyte telomere length and cigarette smoking, dietary and physical variables, and risk of prostate cancer. Aging Cell.

[51] Shiels P.G., McGlynn L.M., MacIntyre A., Johnson P.C.D., Batty G.D., Burns H., Cavanagh J., Deans K.A., Ford I., McConnachie A. (2011). Accelerated Telomere Attrition Is Associated with Relative Household Income, Diet and Inflammation in the pSoBid Cohort. PLoS ONE.

[52] Astuti Y., Wardhana A., Watkins J., Wulaningsih W. (2017). Cigarette smoking and telomere length: A systematic review of 84 studies and meta-analysis. Environ. Res..

[53] Gardner M., Bann D., Wiley L., Cooper R., Hardy R., Nitsch D., Martin-Ruiz C., Shiels P., Sayer A.A., Barbieri M. (2014). Gender and telomere length: Systematic review and meta-analysis. Exp. Gerontol..

[54] Glasgow R.E., Ory M.G., Klesges L.M., Cifuentes M., Fernald D.H., Green L.A. (2005). Practical and Relevant Self-Report Measures of Patient Health Behaviors for Primary Care Research. Ann. Fam. Med..

